# The Eucalyptus in Ague

**Published:** 1876-12

**Authors:** 


THE EUCALYPTUS IN AGUE.—Dr. John Curnow, of London, reports the following cases in the *Lancet*:

CASE 1. S. S., aged 18, a Norwegian, was admitted May 23, 1876. He had been suffering from intermittent fever for four or five weeks. The attacks were moderately severe and of a well-marked tertian type. An expectant plan of treatment was pursued until June 9th, and during this period the paroxysms recurred on alternate days with the utmost regularity. They began at 10 A.M., reached their acme between 1:30 and 3 P.M., and passed off about 6 P.M.; thus lasting about eight hours. The highest temperature varied from 104.8° to 105.6°. On June 9th the tincture of the eucalyptus globulus was given in one-drachm doses three times daily. The next day, on which another attack was due, his temperature only arose to 100°, and on the 12th to 100.4°; and after this date no further paroxysm occurred during the remainder of his stay in the hospital. On physical examination, a systolic bruit was heard over the apex of the heart, but this was evidently of some standing, and had so far given rise to no symptoms. The splenic dullness was normal.

Case 2. C. O., aged 40, a Dane, was admitted on June 19th, 1876. The attack commenced on June 14th, and was of the ordinary tertian type. The paroxysms were very severe, and extended over nearly twelve hours, on an average. On June 27th, the temperature was carefully taken at short intervals by Mr. Lacy, the house physician. At 10:30 A.M. it was normal; at 11:30 it had risen slightly, and soon after rigors set in; at 12:40 P.M. it was 101.6°; at 2.20 P.M. 105.6°; at 2:40 P.M. it had reached its highest point, 106.4°; at 3 P.M. it had fallen to 105.4°; at 6 P.M. to 101.4°; at 9 P.M. to 100; and at midnight it was still above normal, at 99.2°. The fit on the 29th was quite as severe. On July 1st, just before the next attack was due, the expectant plan of treatment, which had hitherto been pursued, was given up, and the tincture of the eucalyptus exhibited in drachm doses three times a day. The next two paroxysms were much shortened in length, and the temperature did not rise quite so high. On the 5th the dose was increased to two drachms three times daily, and he had his last attack on the next day. He was kept under observation until July 15th, and continued taking the medicine up to this date. The patient's splenic dullness was increased in extent, and the edge could just be felt. He had suffered from an attack of ague nine years before.—*Reporter.*

